# High Resolution Patterning of an Organic–Inorganic Photoresin for the Fabrication of Platinum Microstructures

**DOI:** 10.1002/adma.202101992

**Published:** 2021-08-01

**Authors:** Manuel Luitz, Markus Lunzer, Andreas Goralczyk, Markus Mader, Sagar Bhagwat, Andreas Warmbold, Dorothea Helmer, Frederik Kotz, Bastian E. Rapp

**Affiliations:** ^1^ Laboratory of Process Technology NeptunLab Department of Microsystems Engineering (IMTEK) University of Freiburg Georges‐Köhler‐Allee 103 79110 Freiburg Germany; ^2^ UpNano GmbH Modecenterstraße 22/D6 Vienna 1030 Austria; ^3^ Freiburg Materials Research Center (FMF) University of Freiburg Stefan‐Meier‐Straße 21 79104 Freiburg Germany; ^4^ FIT Freiburg Center of Interactive Materials and Bioinspired Technologies University of Freiburg Georges‐Köhler‐Allee 105 79110 Freiburg Germany

**Keywords:** direct lithography, metal printing, nanofabrication, platinum microstructures, printed electronics, two‐photon lithography

## Abstract

Platinum (Pt) is an interesting material for many applications due to its high chemical resilience, outstanding catalytic activity, high electrical conductivity, and high melting point. However, microstructuring and especially 3D microstructuring of platinum is a complex process, based on expensive and specialized equipment often suffering from very slow processing speeds. In this work, organic–inorganic photoresins, which can be structured using direct optical lithography as well as two‐photon lithography (TPL) with submicrometer resolution and high‐throughput is presented. The printed structures are subsequently converted to high‐purity platinum using thermal debinding of the binder and reduction of the salt. With this technique, complex 3D structures with a 3D resolution of 300 nm were fabricated. At a layer thickness of 35 nm, the patterns reach a high conductivity of 67% compared to bulk platinum. Microheaters, thermocouple sensors as well as a Lab‐on‐a‐Chip system are presented as exemplary applications. This technology will enable a broad range of application from electronics, sensing and heating elements to 3D photonics and metamaterials.

## Graphical abstract

High‐resolution patterning of an organic–inorganic platinum containing photoresin by microlithography and two‐photon polymerization is demonstrated. The polymerized microstructures are debinded and reduced to highly conductive metallic platinum at a resolution of 300 nm. The conductivity of the reduced platinum structures is 67% of the conductivity of bulk platinum.

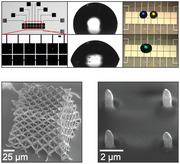

## 1 Introduction

Platinum (Pt) is an important material due to its high electrical conductivity of 9.52 × 10^6^ S m^−1^, its chemical and thermal stability, catalytic activity as well as its biocompatibility.^[^ ^1^, ^2^ ^]^ Microstructured platinum has been used in a variety of fields from electronics, sensors, and microelectromechanical systems (MEMS) to Lab‐on‐a‐Chip (LOC) devices, cochlear implants, and fuel cells.^[^ ^3^, ^4^, ^5^, ^6^, ^7^, ^8^ ^]^ Pt is used for manufacturing of microheaters,^[^ ^9^, ^10^ ^]^ temperature‐,^[^ ^11^, ^12^ ^]^ gas‐,^[^ ^13^ ^]^ flow‐,^[^ ^14^ ^]^ or biosensors ^[^ ^15^, ^16^ ^]^ as well as catalysts for fuel cells^[^ ^7^, ^17^, ^18^ ^]^ and dye sensitized solar cells^[^ ^19^, ^20^, ^21^ ^]^ to name but a few. However, microstructuring of Pt is challenging and usually done by physical vapor deposition (PVD) techniques such as electron beam deposition or sputtering.^[^ ^22^, ^23^ ^]^ These methods require multiple steps such as lithography, lift‐off and etching, which are time consuming and depend on highly specialized equipment as well as clean room facilities.^[^ ^8^, ^9^, ^10^, ^24^ ^]^ Inkjet printing has been widely used in the last decade to structure metallic electrodes using either nanoparticle based or organic–inorganic inks.^[^ ^21^, ^25^ ^]^ However, inkjet printing suffers from low resolution due to the fusion of droplets at low pixel sizes and inhomogeneity due to the coffee‐ring effect, which can occur during the drying process.^[^ ^26^, ^27^, ^28^ ^]^ Furthermore, all mentioned technologies only allow the fabrication of 2.5‐dimensional microstructures. 2.5‐dimensional structures are defined as 2D objects on a *x*/*y*‐plane with a defined expansion in *z*‐direction.^[^ ^29^ ^]^ However, for applications like electronic interconnects, photonics or metamaterials, methods for 3D structuring of Pt on the micrometer to submicrometer scale are highly sought after.^[^ ^30^, ^31^, ^32^, ^33^ ^]^ So far only a few 3D printing methods have been established allowing the fabrication of truly 3D Pt microstructures. Laser induced forward transfer (LIFT) was utilized to fabricate tilted wire‐like structures with a resolution of 10 µm.^[^ ^34^ ^]^ However, overhanging structures are difficult to obtain because of the fact that material is deposited orthogonally to the substrate only and the landing accuracy of the ablated droplets is very low.^[^ ^34^, ^35^ ^]^ Meniscus confined electroplating was employed to produce pillars with diameters of 80 nm and horizontally overhanging Pt interconnects with diameters of 900 nm.^[^ ^36^ ^]^ However, this technique suffers from low fabrication speeds with growth rates of 0.3 µm s^−1^, the nontrivial establishment of a stable liquid bridge under a nanopipette and the necessity of a conductive substrate.^[^ ^36^, ^37^ ^]^ Focused electron/ion beam induced deposition (FEBID/FIBID) was utilized to obtain 3D Pt objects with resolutions of 60 and 110 nm, respectively.^[^ ^38^, ^39^ ^]^ The low purity of the resulting metal structures is a well‐known issue in FEBID/FIBID, due to the use of organic precursors, which lead to high amounts of organic contaminants in the final Pt parts.^[^ ^40^ ^]^ To achieve high purity deposition from gaseous precursors, FEBID/FIBID writing speeds must be in the range of tens of nm s^−1^ which makes structuring micrometer‐scale objects very time‐consuming.^[^ ^40^ ^]^ A comparison of microfabrication techniques of platinum is summarized in Table S1 (Supporting Information). As can be seen, faster methods for high‐resolution 3D micro and nanostructuring of platinum are highly desirable.

Two‐photon lithography (TPL) is a versatile technique for high‐resolution 3D structuring.^[^ ^41^, ^42^ ^]^ Compared to the aforementioned 3D structuring techniques, TPL reaches a higher throughput with writing speeds up to 8000 mm s^−1^.^[^ ^43^ ^]^ Recently it was shown that organic–inorganic photoresins can be structured by TPL to form complex 3D nanoarchitected nickel and zinc oxide, which can be converted to their respective metal and metal oxides via heat treatment.^[^ ^44^, ^45^ ^]^ By exploiting the shrinkage of the printed parts, Ni and ZnO objects with beam sizes of 25–100 nm (Ni) and 250 nm (ZnO) were fabricated. So far, the choice of materials, which can be fabricated using photocurable organic–inorganic photoresins is limited and inaccessible for microstructuring of platinum.

In this work, we demonstrate a lithography‐based process to platinum microstructuring using a photocurable organic–inorganic Pt‐based photoresin. This process allows the fabrication of highly pure Pt electrode patterns and Pt layers of thicknesses between 35 and 60 nm, with an electrical conductivity of 6.32 × 10^6^ S m^−1^. This is 67% compared to bulk Pt metal, and a high value for such platinum nanolayers.^[^ ^46^ ^]^ We further show that these organic–inorganic photoresins can be 3D structured at a writing speed of 100 mm s^−1^ by means of TPL, giving a final resolution of 330 nm after thermal reduction. The structured organic–inorganic polymer components are converted to pure Pt(0) metal by thermal debinding of the polymeric binder matrix and the thermal reduction of the Pt(II) salt.

**Figure 1:**
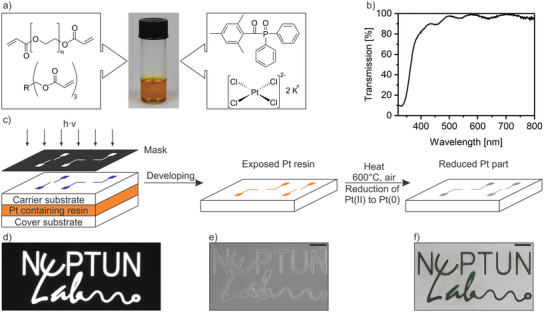
Structuring platinum using organic–inorganic photoresins. a) Preparation of the Pt containing organic–inorganic photoresin by mixing potassium tetrachloroplatinate, water, PEGDA, Genomer 7311 and TPO. b) UV–Vis spectrograph of the platinum resin without the photoinitiator, showing a transmission of 87% at 415 nm and 95% at 780 nm. c) Schematic of the Pt structuring process by means of direct mask‐based lithography. The resin is polymerized at a wavelength of 415 nm. Nonpolymerized resin is removed using ethanol and water. The organic–inorganic polymer is converted to Pt metal using thermal debinding and reduction. d) Image of the photomask utilized to fabricate the NeptunLab logo. e) Micrograph of the respective organic–inorganic polymer of the NeptunLab logo (scale bar: 500 µm). f) Reduced platinum structure derived from the organic–inorganic polymer in e) (scale bar 500 µm).

The direct lithography process is shown in **Figure** **1**. The Pt containing organic–inorganic photoresin was prepared by dissolving 25 wt% of K_2_PtCl_4_ in 75 wt% of an acrylic binder matrix. The mixture was treated with a sonotrode to break down the Pt salt crystals into a fine powder and subsequently stirred until the Pt precursor was dissolved completely. For high‐resolution structuring, the optical transparency at the wavelength of polymerization must be high.^[^ ^47^ ^]^ To demonstrate the high transparency of the organic–inorganic photoresin, the UV–vis spectrum of the Pt/acrylate mixture without the initiator was measured (see Figure 1b; and Figure S1, Supporting Information for the absorption spectra of the organic–inorganic photoresin and PI). The organic–inorganic photoresin displays a high transparency of 87% at 415 nm and of 95% at 780 nm, which makes it suitable for optical lithography (415 nm) as well as TPL (780 nm). To demonstrate structuring at 415 nm, the developed organic–inorganic photoresin was blended with a photoinitiator and locally cured using mask‐based optical lithography via radical polymerization. The Pt ion rich organic–inorganic photoresin is crosslinked to a Pt ion rich polymer, where the Pt ions are entrapped and homogeneously distributed in the polymeric network. A schematic of the process is shown in Figure 1c. The organic–inorganic photoresin is casted on a fused quartz carrier substrate and enclosed with a fused quartz cover substrate to avoid oxygen inhibition (see also Figure S2, Supporting Information for the general setup for lithography). The photomask was placed on top of the carrier substrate and the resin was locally cured at a wavelength of 415 nm and an exposure dose of 90 mJ cm^−2^. Subsequently, the cover substrate was removed and the nonpolymerized resin was developed in a 1:1 v:v mixture of H_2_O and EtOH for 10 min. The resulting organic–inorganic polymer structure was converted into Pt by thermal treatment at 600 °C, leading to the decomposition and removal of the binder matrix and thermal reduction of the Pt(II) salt to Pt(0). The temperature of 600 °C was chosen based on the decomposition of K_2_PtCl_4_, where two of the four chlorine atoms are removed at 370 °C forming PtCl_2_ and the remaining two chlorine atoms are removed at around 600 °C forming metallic Pt.^[^ ^48^ ^]^ In Figure 1d–f, an exemplary platinum structure created by this process using the logo of the NeptunLab is shown.

**Figure 2:**
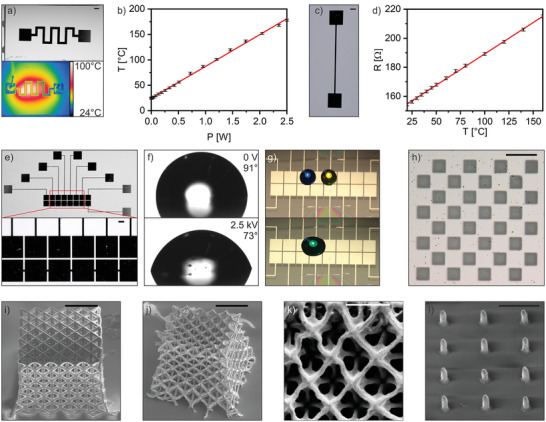
Microstructuring of platinum using lithography and TPL. a) Structured platinum microheater with a platinum layer thickness of 35 nm with an IR image of the heater at 100 °C by applying 1.2 W (scale bar 2 mm). b) Calibration curve of the microheater shown in a). c) Temperature sensor based on a printed Pt100 thermocouple with a platinum layer thickness of 60 nm (scale bar 2 mm). d) Calibration curve of the temperature sensor shown in c) demonstrating a linear dependency of resistivity over temperature. e) Image of an EWOD device for DMF with a platinum layer thickness of 30 nm. The inset shows a micrograph of the fabricated electrodes (scale bar 500 µm). f) Contact angles of a water droplet at 0 V and at 2.5 kV. g) DMF application using the printed platinum electrode array showing the mixing of a blue and yellow droplet, respectively, to a green droplet by applying 2.5 kV. h) Fabricated platinum chessboard with a square size of 40 µm using maskless lithography and a layer height of 30 nm (scale bar 100 µm). i) SEM image of an octet lattice structure printed by TPL. The beams exhibit a diameter of 10 µm. (scale bar 100 µm. j) SEM image of the reduced octet lattice from i). The beam diameter after thermal treatment is 2 µm (scale bar 50 µm). k) Top view of a smaller reduced octet lattice with a beam diameter of 300 nm (scale bar 3 µm). l) Platinum nanopillars after thermal treatment. The pillars have a diameter of 700 nm and a height of 1.5 µm (scale bar 5 µm).

Using this approach, we fabricated a number of platinum microstructures, demonstrating the versatility of this fabrication technique (see **Figure** **2**). Integrated heaters and temperature sensors are of great importance for MEMS and LOC devices for applications like on‐chip polymerase chain reaction (PCR), where temperature control is crucial.^[^ ^49^, ^50^ ^]^ We have fabricated a heating element (Figure 2a) with a lateral resolution of 1 mm and a layer thickness of 40 nm. The device's temperature as a function of the applied power was measured using an infrared (IR) camera (see Figure 2b). The resulting plot displays a linear correlation, which allows a precise temperature control. For measuring temperature, we fabricated a Pt 100 thermocouple with a lateral resolution of 500 µm and a layer thickness of 60 nm (see Figure 2c). The calibration curve of the thermocouple is shown in Figure 2d, showing a linear correlation between the resistivity and temperature over a temperature range from 20 to 160 °C, which allows the utilization of this resistor as a temperature sensor. To demonstrate the fabrication of electrode arrays, we chose a mask‐design for digital microfluidics (DMF, see Figure 2e–g). DMF is based on the concept of electrowetting on dielectrics (EWOD) and is a widely used method for manipulation of liquid droplets on an array of electrodes for applications like chemical reactions, immunoassays or clinical diagnostics.^[^ ^51^, ^52^, ^53^ ^]^ The printed and reduced Pt electrodes were coated with a dielectric layer of 10 µm of parylene C. By applying 2.5 kV between two adjacent electrodes, the contact angle of a water droplet changed from 91° to 73° (see Figure 2f). Figure 2g shows the functional DMF device displaying the mixing of two separate colored water droplets. We further demonstrated maskless lithography based on a digital mirror device to allow for flexible 2.5‐dimensional platinum structuring, without the need for mask fabrication as can be seen from the chessboard structure in Figure 2h.^[^ ^54^ ^]^ High‐resolution 3D microstructuring of the organic–inorganic photoresin was achieved by means of TPL using the commercially available high‐resolution 3D printing system NanoOne from UpNano. Using TPL we fabricated platinum 3D octet lattices as well as pillar arrays with a resolution down to 300 nm after thermal treatment (see Figure 2i–l). The structures were printed with a very fast writing speed of 100 mm s^−1^.

**Figure 3:**
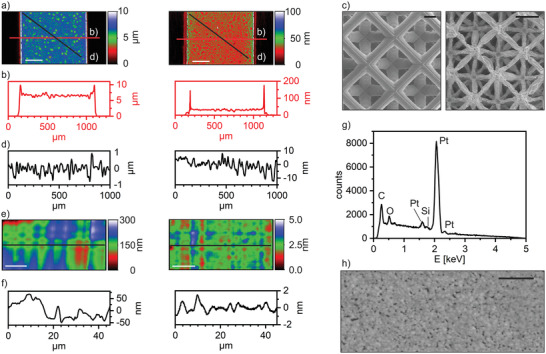
Characterization of the structured platinum. a,b) Shrinkage analysis using WLI of platinum structures fabricated by means of mask‐based lithography on top of a fused quartz substrate. The polymeric organic–inorganic structure is displayed on the left side and its reduced form on the right side. The measured profile is displayed in b). The polymeric organic–inorganic structure had an average height of 6.5 µm shrinking by a factor of 186 to an average height of 35 nm during sintering and reduction. The lateral dimensions of 944 µm remain unchanged due to the pinning on the substrate (scale bar 500 µm). c) Shrinkage analysis using SEM images of free‐standing platinum structures fabricated by means of TPL. The polymeric organic–inorganic structure (left) shrank from 9.9 ± 0.2 to 1.9 ± 0.2 µm in its reduced Pt form (right) by a factor of 5.2 (scale bar 10 µm). d) Surface analysis using WLI of Pt structures fabricated by means of mask‐based lithography on top of a fused quartz substrate. The organic–inorganic polymer structure (left) had a surface roughness of *R*_a_  =  209 nm and its reduced form (right) of *R*_a_  =  2.7 nm (scale bar 500 µm). e,f) Surface analysis using WLI of platinum structures fabricated by means of TPL. The polymerized organic–inorganic structure (left) had a *R*_a_  =  30 nm and its reduced form (right) of *R*_a_  =  0.3 nm (scale bar 10 µm). g) EDX spectrograph showing the high‐purity of the reduced platinum structures with 96.8 wt% of platinum, 1.6 wt% carbon, 1.3 wt% oxygen, and 0.3 wt% silicon. h) SEM image of a reduced Pt film. The sum of the pore area in relation to the total area revealed a porosity of 3.5%. The pores displayed an average Feret diameter of 50 ± 30 nm (scale bar 1 µm).

We have investigated the shrinkage of the Pt containing organic–inorganic polymer during the thermal heat treatment comparing free‐standing structures and structures printed on top of a nonshrinking substrate. **Figure** **3** shows the analysis of the structures using white light interferometry (WLI). Figure 3a,b shows the shrinkage of a structure, which was fabricated and heat treated on top of a fused quartz glass substrate. As can be seen, debinding and reducing on top of a nonshrinking substrate results in a highly anisotropic shrinkage. During the thermal treatment, the component shrinks in the z‐direction by a factor of 186. The thickness decreased from 6.5 µm to 35 nm, while the lateral dimensions of 944 µm did not change from the structured organic–inorganic polymer state to the reduced platinum state. The free‐standing structures fabricated via TPL on the other hand showed a near isotropic shrinkage. The beam size of the polymerized octet lattice in Figure 3c was 9.9 ± 0.2 µm in the organic–inorganic polymer state and after thermal treatment the reduced Pt object had a beam size of 1.9 ± 0.2 µm demonstrating a shrinkage by a factor of 5.2 (Tables S3 and S4, Supporting Information for the shrinking analysis). The beams of the octet lattice further showed slight warping and folding after the heat treatment.

The surface roughness of the printed organic–inorganic polymer and the reduced platinum was further characterized using WLI. The organic–inorganic polymer structured using direct mask‐based lithography showed a surface roughness *R*_a_ of 209 nm and the reduced part showed a *R*_a_ of 2.7 nm (see Figure 3d). The test specimens fabricated with TPL showed a *R*_a_ of 30 and 0.3 nm in the polymerized state and reduced state, respectively (see Figure 3e,f). The line edge roughness, aspect ratios, and line edge perpendicularities of samples before and after thermal treatment were investigated (Tables S5 and S7 and Figures S4 and S5, Supporting Information). The line edge roughness increased slightly from the polymerized state to the reduced state from 1.7 ± 0.3 to 15.4 ± 13.2 µm. The aspect ratio decreased from 0.014 ± 0.001 to 0.06 × 10^–^^3^ ± 0.01 × 10^−3^. The line edge perpendicularity decreased from 88.4 ± 0.5° to 81.3 ± 1.2°, respectively. To analyze the purity of the printed platinum, the reduced structures were characterized using energy dispersive X‐ray spectroscopy (EDX) (see Figure 3g). The EDX analysis revealed that the reduced parts contained 96.8 wt% of platinum and 3.2 wt% contaminants consisting of 1.6 wt% carbon, 1.3 wt% oxygen, and 0.3 wt% silicon. This purity degree is very high, especially compared to 3D structuring methods like FEBID reaching platinum purities of around 70 wt% for gas phase depositions and 90% for liquid phase depositions.^[^ ^55^, ^56^ ^]^ Scanning electron microscopy (SEM) was performed to analyze the porosity of the reduced platinum. Here, the porosity is defined as the sum of the area of the pores in relation to the whole area. The SEM image in Figure 3h shows a platinum layer with a porosity of 3.5% and an average Feret diameter of 50 ± 30 nm of the pores. To determine the electrical conductivity of the reduced platinum parts, the resistivity of the platinum heater from Figure 2a was measured and the conductivity was calculated. The reduced platinum part reached a conductivity of 6.32 × 10^6^ S m^−1^, which is 67% of the conductivity of bulk platinum. Considering the fact that these are thin layers of only 35 nm, this is a high value compared to platinum layers fabricated by PVD processes, where a comparable platinum layer thickness of 28 nm reaches about 23% of conductivity of bulk platinum.^[^ ^46^ ^]^ Inkjet printed Pt layers with a layer thickness of 75 nm and Pt layers with a thickness of 150 nm fabricated by means of FEBID, have been reported to reach conductivities of about 16% and less than 1%, respectively, compared to bulk platinum.^[^ ^27^, ^55^ ^]^

In summary, we demonstrated a novel method to shape highly conductive platinum using microlithography techniques as well as TPL. We were able to fabricate ultrathin electrode patterns of tens of nanometers using standard additive manufacturing process workflows. The fabricated electrode patterns were demonstrated successfully in practical applications such as for the design of a microheater, a Pt100 thermocouple, and DMF. By utilizing TPL, we were able to fabricate freestanding nanopillars as well as complex 3D Pt microstructures with a resolution of 300 nm. Such small platinum pillars and scaffolds will find use in various engineering applications including metamaterials or catalysis, where high surface areas and the physicochemical properties of platinum are highly desirable.

## 2 Experimental Section

### Materials

2‐propanol (IPA) and ethanol (EtOH) were purchased from Carl Roth (Germany). Polyethylene glycol diacrylate (PEGDA 575) with an average molar mass of 575 g mol^−1^, trimethylolpropane triacrylate (TMPTA), toluene, diphenyl‐(2,4,6‐trimethylbenzoyl)‐phosphine oxide (TPO), hydroquinone (HQ) and 4,4'‐bis(diethylamino)benzophenone (Michler's ethyl‐ketone, MEK) were purchased from Sigma‐Aldrich (Germany). Potassium tetrachloroplatinate (K_2_PtCl_4_), 3‐methacryloxypropyl‐dimethylchlorosilane (MACS), 1H, 1H, 2H, 2H‐perfluorooctyldimethylchlorosilane (PFODMClS) were purchased from abcr (Germany). Genomer 7311 was kindly provided by Rahn Chemicals (Switzerland). Fused quartz glass slides (50.8 × 25.4 × 1 mm^3^, and 76.2 × 50.8 × 1 mm^3^) were purchased from Plano (Germany). Borosilicate glass substrates (10 × 10 × 5.5 mm^3^) for TPL were provided by UpNano (Austria).

### Resin Preparation

The binder matrix of the Pt containing organic–inorganic photoresin for direct mask‐based lithography consisted of 80 vol% PEGDA 575, 10 vol% of the water soluble monomer Genomer 7311 and 10 vol% of water. The monomers were mixed, 25 wt% of K_2_PtCl_4_ was added to 75 wt% binder matrix and the mixture was treated with an ultrasonic lance for 5 min (UP200St, Hielscher Ultrasonics, Germany) and stirred for 2 d. After the Pt salt was completely dissolved, the resin was filtered using a syringe filter with a mesh size of 0.45 µm. Then, 0.5 wt% of the photoinitiator (PI) TPO was added to the organic–inorganic photoresin and sonicated until it was completely dissolved. For the maskless lithography 0.25 wt% of TPO and 0.1 wt% of HQ were added. For the Pt‐containing organic–inorganic photoresin for TPL, the amount of crosslinker and the type of PI was adapted. Here, the binder matrix consisted of 70 vol% PEGDA 575, 10 vol% TMPTA, 10 vol% of Genomer 7311, and 10 vol% of water. 1 wt% of MEK was added as PI and the mixture was sonicated until the solid was completely dissolved.

### Surface Functionalization

The fused quartz substrates were treated for 30 min in acidic methanol (methanol:HCl, 1:1 v:v). Subsequently, the substrates were washed with IPA and deionized (DI) water and dried with nitrogen. Subsequently, the carrier substrates were immersed in a 100 × 10^−3^ m solution of MACS in dry toluene and the cover substrates were immersed in a 100 × 10^−3^ m solution of PFODMClS in dry toluene for 60 min. The substrates were again washed with IPA and DI water and subsequently dried using nitrogen.

The 10 × 10 × 5 mm^3^ borosilicate glass substrates (UpNano) for TPL were functionalized using 3‐ (trimethoxysilyl)propyl methacrylate following a modified procedure from literature.^[^ ^57^ ^]^ A solution was prepared by mixing 50:48:0.3:2 v:v deionized water, ethanol, glacial acetic acid, and 3‐(trimethoxysilyl)propyl methacrylate. The solution was stirred for 15 min before substrates were submersed. After 30 min of surface treatment, the substrates were rinsed with deionized water twice and then dried in a drying chamber (50 °C) for ≈1 h.

### Lithography

The image exposure step was performed using a high‐pressure mercury lamp Superlite 400 (Lumatec, Germany). To perform lithography, the resin was drop casted onto the carrier substrate. Then, the cover substrate was placed on top and both substrates were pressed together using two clips (see Figure S1, Supporting Information). The photomask was placed on top of the carrier substrate. UV exposure was done with an exposure dose of 90 mJ cm^−2^ at a wavelength of 415 nm. After the exposure, the cover substrate was removed and the carrier substrate was washed with a 1:1 v:v mixture of DI water and EtOH. Subsequently the samples were immersed in the same developer for 10 min.

### EWOD Device Manufacturing

The electrode pattern for the EWOD device was manufactured using the lithography technique. The electrode array was coated with a 10 µm thick dielectric layer of parylene C. The parylene C layer was deposited on the sample using a PDS 2010 LABCOATER 2 parylene deposition system (Specialty Coating Systems, United Kingdom).

### Two‐Photon Lithography

Two‐photon lithography was performed using a NanoOne high‐resolution printing system (UpNano GmbH, Austria) equipped with a 20x oil immersion objective (NA 0.85, UPLSAPO20XO, Olympus) in Vat mode. Here, the laser (80 MHz repetition rate, 90 fs pulse length, 780 nm center wavelength, 1595.9 GW cm^−2^ intensity at focus, Equation (S1) (Supporting Information) for the calculation of intensity at focus)^[^ ^58^ ^]^ is focused through a high precision cover glass into a material vat containing the resin and maintained at a constant height above the glass. For layer‐wise 3D structuring, the laser is scanned along the XY‐plane by a galvanometer scanner and the objective together with the vat is lowered along the *z*‐axis using a piezo stage. All microstructures were fabricated with a laser power of 35 mW at a scanning speed of 100 mm s^−1^. *x*,*y*‐planes were sliced alternately in *x*‐ and *y*‐direction using a line distance (hatch, ∆*xy*) of 0.20 µm and a slicing distance (∆*z*) of 1 µm.

### Heat Treatment

Thermal debinding of the binder matrix and reduction of platinum salt was performed using an ashing furnace of the type AAF (Carbolite/Gero, Germany). The entire protocol for thermal treatment can be found in in Table S2 (Supporting Information).

### Material Characterization

Dimensions and surface roughness were measured using a WLI of type NewView 9000 (Zygo, USA). Scanning electron micrographs were taken using an ultrahigh resolution focused ion beam SEM of type Scios 2 DualBeam (Thermo Fisher Scientific, Germany). EDX spectra were taken using an Octane Elite EDS System (EDAX, Germany). The electrical resistivities of the reduced Pt parts were measured using a multimeter of type 34401A Digit Multimeter (Keysight, Germany). The contact angles of water droplets with a drop size of 2 µL on the EWOD device were measured using an OCA15EC (Dataphysics, Germany). The optical transparency and absorption spectra of the organic–inorganic photoresin were measured using a UV–vis spectrophotometer of type Evolution 201 (Thermo Scientific, Germany) at a sample thickness of 10 µm. The absorption spectra of the utilized photonitiators were measured in acetonitrile at a concentration of 0.01 mg mL^−1^ through a 1 cm wide fused silica cuvette. The porosities and pore sizes were determined via digital image analysis by thresholding and subsequent calculation of the resulting Feret diameters using ImageJ (see Figure S3, Supporting Information for the digital image analysis). The line edge roughness was calculated using digital image processing using ImageJ (see Figure S4, Supporting Information for the digital image analysis and Table S7 (Supporting Information) for the line edge roughness). The measurements for the calibration of the heating device and the Pt100 thermocouple were repeated three times. The thermogravimetric analysis was performed using a simultaneous thermal analyzer of type STA 449 F5 Jupiter (Netzsch, Germany). The samples were heated with 3 °C min^−1^ from room temperature to 600 °C and dwelled for 1 h (see Figure S6, Supporting Information for the thermogravimetric analysis of the organic–inorganic photoresin).

## Conflict of Interest

The authors declare no conflict of interest.

## Supporting information

Supporting Information

Supplemental Video 1

## Data Availability

The data that supports the findings of this study are available in the supplementary material of this article.
