# Supplementary material for: High Resolution Patterning of an Organic–Inorganic Photoresin for the Fabrication of Platinum Microstructures
Source: Adv Mater. 2021 Aug 1;33(37):2101992. doi: 10.1002/adma.202101992 (PMC11469048; doi:10.1002/adma.202101992)
Supplement: Supplementary file 1 — Supporting Information [file ADMA-33-2101992-s002.pdf]

© 2021 Wiley-VCH GmbH

ADVANCED MATERIALS

# Supporting Information

for *Adv. Mater.,* DOI: 10.1002/adma.202101992

# High Resolution Patterning of an Organic–Inorganic Photoresin for the Fabrication of Platinum Microstructures

*Manuel Luitz, Markus Lunzer, Andreas Goralczyk, Markus Mader, Sagar Bhagwat, Andreas Warmbold, Dorothea Helmer, Frederik Kotz,* and Bastian E. Rapp*

Supporting Information

## High Resolution Patterning of an Organic-Inorganic Photoresin for the Fabrication of Platinum Microstructures

*Manuel Luitz, Markus Lunzer, Andreas Goralczyk, Markus Mader, Sagar Bhagwat, Dorothea Helmer, Frederik Kotz, Bastian E. Rapp*

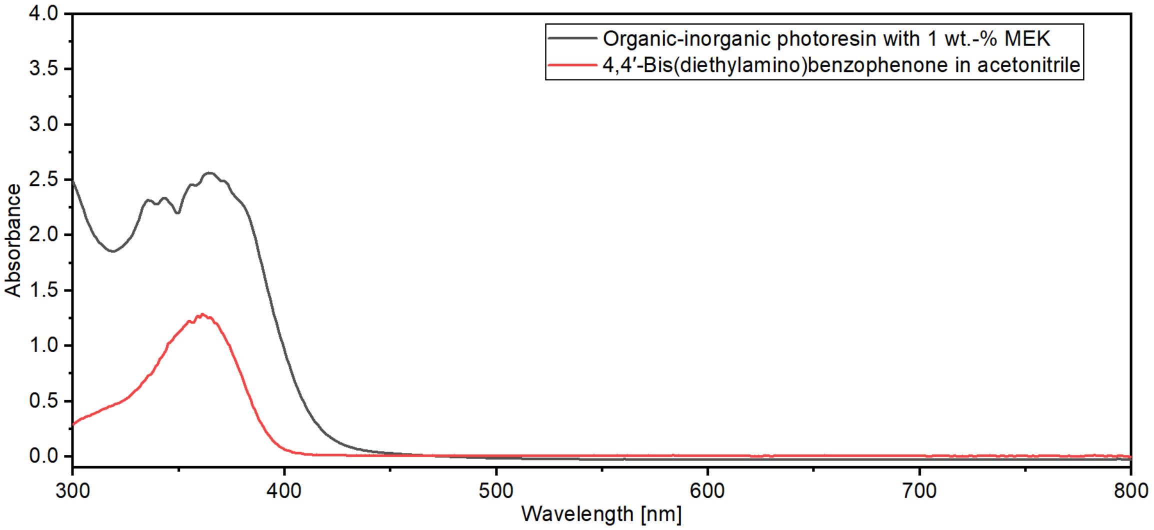


**Figure S1:** Absorption spectrum of the organic-inorganic photoresin with 1 wt.-% of 4,4-bis(diethylamino) benozophenone (MEK) and MEK in acetonitrile at a concentration of 0.01 mg $mL^{-1}$.

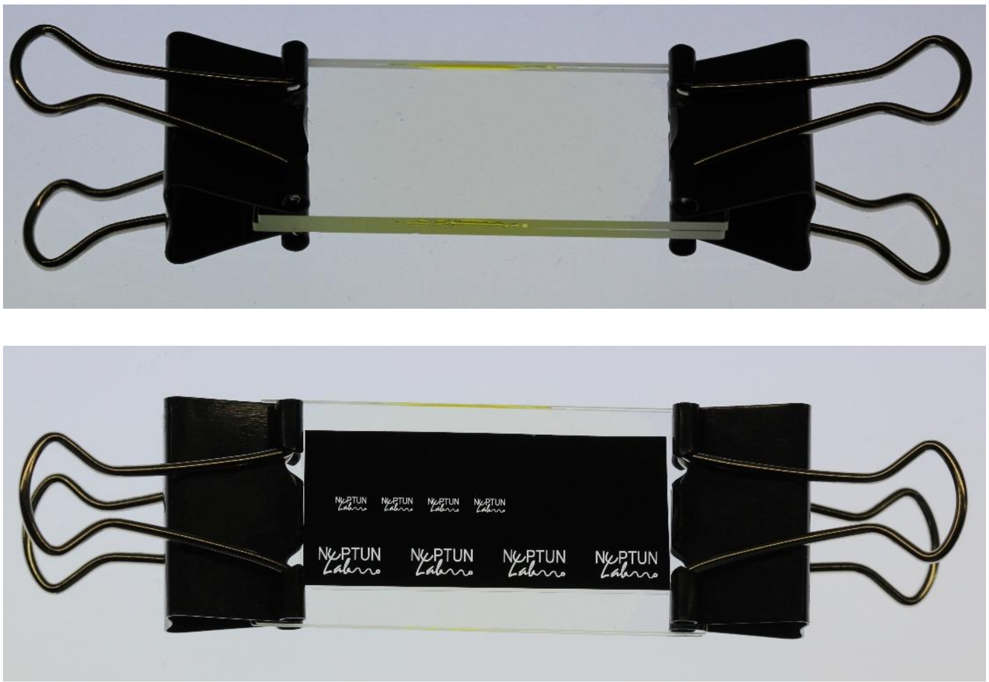

**Figure S2. Set up for the mask-based optical lithography.** The resin was drop-cast onto a fused quartz substrate and covered with a second fused quartz substrate. The substrates were pressed together using binder clips. Subsequently, the respective photomask was placed on top of the carrier substrate prior to UV exposure.

The intensity at focus of the laser was calculated using Equation S1.

$$I_{Peak} = 0.88\frac{P_{avg}\pi(NA)^2}{R\tau\lambda^2} = 1595.9\frac{GW}{cm^2} \qquad \text{S1}$$

| | |
|---|---|
| $I_{Peak}$ | Intensity at focus [GW cm$^{-2}$] |
| $P_{avg}$ | Laser power [W] |
| $NA$ | Numerical aperture |
| $R$ | Repetition rate [Hz] |
| $\tau$ | Pulse length [s] |

The factor 0.88 results in the assumption that the focal spot is Gaussian and the pulse shape is sech$^2$ (squared hyperbolic secant).[1]

**Table S1:** Comparison of relevant methods for microstructuring of platinum.

| Method | Resolution | Speed | 3D capability | Contaminants | Literature |
|---|---|---|---|---|---|
| Inkjet printing | 20 µm | n.a. | - | n.a. | [2,3] |
| Laser induced forward transfer | 10 µm | n.a. | + | n.a. | [4] |
| Meniscus confined electroplating | 0.9 µm | 0.3 µm $s^{-1}$ | ++ | n.a. | [5] |
| Focused electron/ion beam induced deposition | 0.02 µm | 0.026 - 0.034 µm $s^{-1}$ | +++ | 10 – 30 wt.-% | [6–9] |
| Two-photon lithography | 0.3 µm | 100 mm $s^{-1}$ | +++ | 3 wt.-% | This work |

**Table S2.** Protocol for thermal treatment.

| Temperature [°C] | Heating rate [°C/min] | Dwelling time [min] |
|---|---|---|
| 600 | 1 | 60 |
| 25 | 5 | - |

**Table S3.** Shrinkage analysis of free-standing organic-inorganic polymer structures to reduced Pt structures.

| Beam number | Diameter in the polymeric form [µm] | Diameter in the reduced Pt form [µm] |
|---|---|---|
| 1 | 10.119 | 2.256 |
| 2 | 9.996 | 1.741 |
| 3 | 9.872 | 2.15 |
| 4 | 9.872 | 1.502 |
| 5 | 10.243 | 1.831 |
| 6 | 9.626 | 2.164 |
| 7 | 9.996 | 2.042 |
| 8 | 9.872 | 1.831 |
| 9 | 9.502 | 2.001 |
| 10 | 9.872 | 1.943 |
| Mean value [µm] | 9.897 ± 0.216 | 1.946 ± 0.227 |

The shrinking characteristics of the parts are largely dependent on the shrinking characteristics of the supporting substrate. When a sample is produced on top of a non-shrinking substrate (which was the case for optical and maskless lithography, see Figure 2 a/c/e/h) such as a surface modified fused quartz, the organic-inorganic polymer is fully pinned to the surface and thus cannot shrink isotropically. The sample will then only shrink in z-direction. However, for a free-standing structure, such as the octet lattice (see Figure 2 i/j), the object is printed on pedestals at the corners of the octet lattice cube. These pedestals are the anchor points of the octet lattice to the substrate and the object is point pinned to the substrate. The images of Figure 2 i/j show that during the heat treatment, the object does not shrink in x/y direction at these anchor points. Above the anchor points the object shrinks nearly isotropically in x-, y- and z-direction. Since the amount of platinum precursor was the same for both organic-inorganic polymers (pinned versus free-standing), the samples have to shrink in equal amounts. To quantify the shrinking behaviour, we measured the dimensions of the objects in the polymeric state and thermally reduced state and calculated the linear shrinking

factor with Equation S2.

$$Linear\ shrinking\ factor = \frac{Dimension\ after\ printing}{Dimension\ after\ heat\ treatment} \quad S2$$

The pinned structures shrink only in the z-direction by a factor of 186 (shrinking in one dimension) from the organic-inorganic polymer state to the reduced metallic state. The point pinned structures shrink nearly isotropically by a factor of 5.2 (shrinking in all three dimensions) (see Table S4).

**Table S4:** Comparison of the shrinking characteristics of full surface pinned structures versus point pinned structures.

| Type | Dimensions after printing | Dimensions after heat treatment | Linear shrinking factor |
|---|---|---|---|
| Full surface pinning | 6.5 µm[a] | 35 nm[a] | 186 |
| Point pinning | 9.9 µm[b] | 1.9 µm[b] | 5.2 |

[a] Thicknesses of the exposed organic-inorganic polymer versus the reduced part fabricated by optical lithography.
[b] Diameter of the beams of the octet lattice structure in the polymeric state versus the reduced state.

The surface roughness ($R_a$) of the samples are different, because they were manufactured with different techniques and set ups. In general, the $R_a$ prior to heat treatment influences the $R_a$ of the final heat-treated sample: A smoother polymerized surface results in a smoother platinum structure, while rough polymerized surfaces resulted in rough platinum structures. The samples which were manufactured by means of direct optical lithography (see Figure 1c) were sandwiched between two fused quartz slides (carrier and cover substrate), while the samples which were made by means of TPL were written inside the organic-inorganic photoresin medium without the need of a cover substrate (see Figure 2l). Due to the different set ups, there are dissimilar surface characteristics during polymerization and the resulting surfaces display different roughness (see Table S5). For the optical lithography approach, there is surface wetting of the photoresin to the cover substrate and the liquid/solid boundary surface. For the TPL there is no cover substrate and only liquid/liquid boundary surfaces.

**Table S5:** Comparison of the surface roughness of samples manufactured by optical lithography versus TPL.

| Manufacturing technique | $R_a$ prior to heat treatment [nm] | $R_a$ after heat treatment [nm] |
|---|---|---|
| Optical lithography | 209 | 2.7 |
| TPL | 30 | 0.3 |

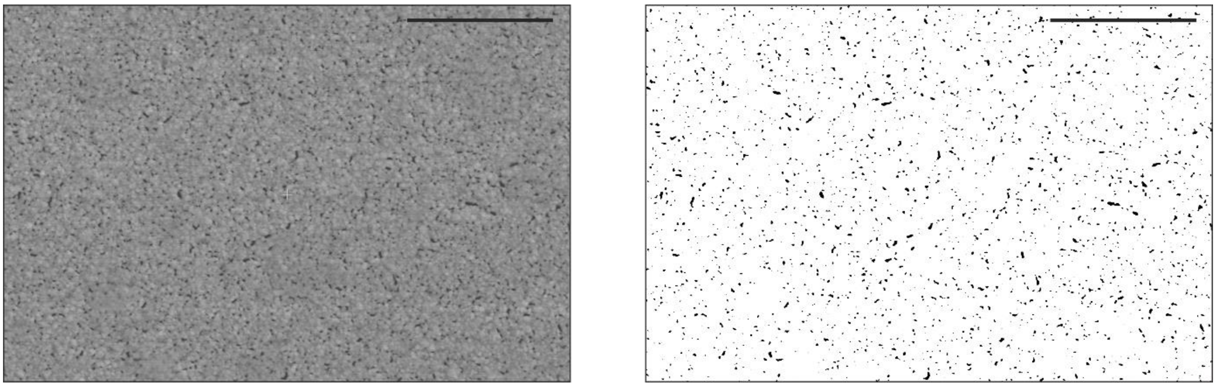

**Figure S3.** Example of a SEM image of a reduced platinum film fabricated via mask-based lithography (left) and the resulting image after using thresholding to determine the pore sizes and porosity (scale bar 3 µm).

**Table S6.** Pore size and porosity analysis via digital image analysis.

| Sample number | Mean Feret diameter [nm] | Porosity [%][a] |
|---|---|---|
| 1 | 49.11 ± 28.66 | 2.96 |
| 2 | 48.82 ± 28,27 | 3.57 |
| 3 | 53.33 ± 31.47 | 4.42 |

[a] Sum of total pore area in relation to total area.

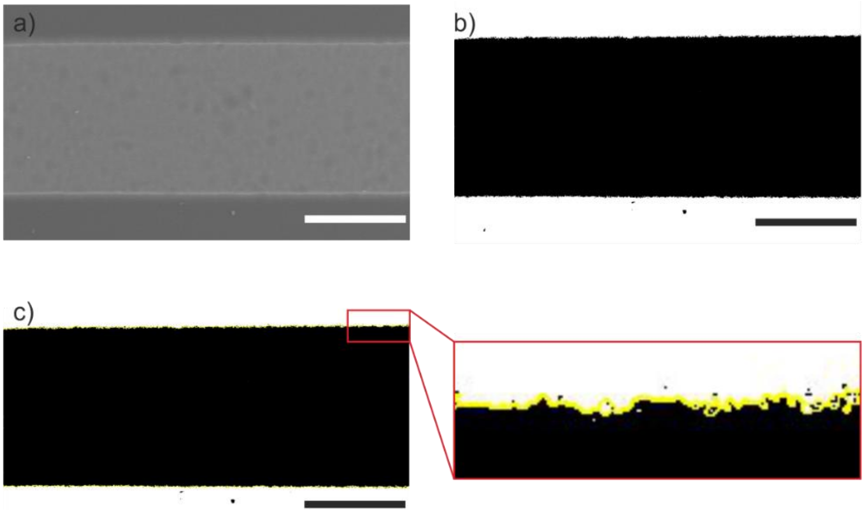


**Figure S4:** Example of the process for the line edge roughness characterization. a) Image of a 470 µm wide conductor path measured by means of SEM and b) after thresh holding. c) Processed image for the determination of the line edge roughness. The yellow line (see inset) was fitted around the line edge (scale bar 300 µm).

**Table S7:** Line edge roughness, line edge perpendicularity and aspect ratios of organic-inorganic polymer samples versus thermally reduced samples. The line edge perpendicularity was determined by calculating the angle between the substrate and the sample. The angles of both sides are given in the table.

| Sample | Linewidth [µm] | Line edge roughness prior to thermal treatment [µm] | Line edge roughness after thermal treatment [µm] | Aspect ratio prior to thermal treatment | Aspect ratio after thermal treatment | Line edge perpendicularity prior to thermal treatment | Line edge perpendicularity after thermal treatment |
|---|---|---|---|---|---|---|---|
| 1 | 475 | 1.8 | 18.5 | 0.015 | $0.07 \cdot 10^{-3}$ | 88.2 / 88.5 | 82.9 / 80.0 |
| 2 | 469 | 1.7 | 8.7 | 0.013 | $0.04 \cdot 10^{-3}$ | 88.3 / 89.2 | 82.2 / 79.8 |
| 3 | 470 | 1.3 | 1.9 | 0.014 | $0.06 \cdot 10^{-3}$ | 87.7 / 88.2 | 81.2 / 81.8 |
| 4 | 476 | 2.1 | 32.4 | 0.015 | $0.08 \cdot 10^{-3}$ | 88.2 / 88.5 | 81.2 / 81.5 |

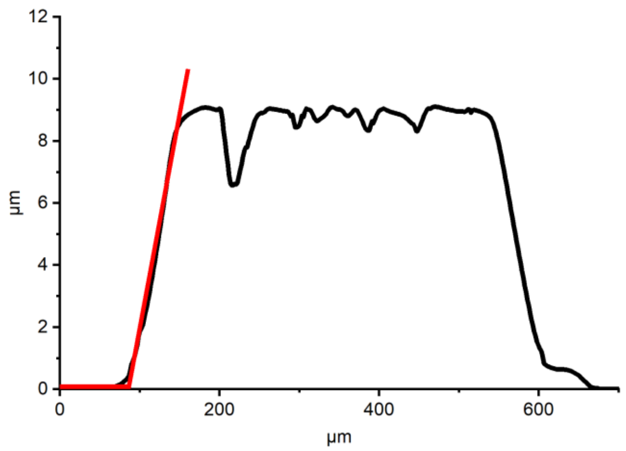


**Figure S5:** Profile of a polymerized organic-inorganic part measured by WLI. The angle between the structure and the substrate (see red lines) was calculated to determine the line edge perpendicularity of the samples. The line edge perpendicularity decreased from 88.4 ± 0.5 ° in the polymeric state to 81.3 ± 1.2 °in the thermally reduced state.

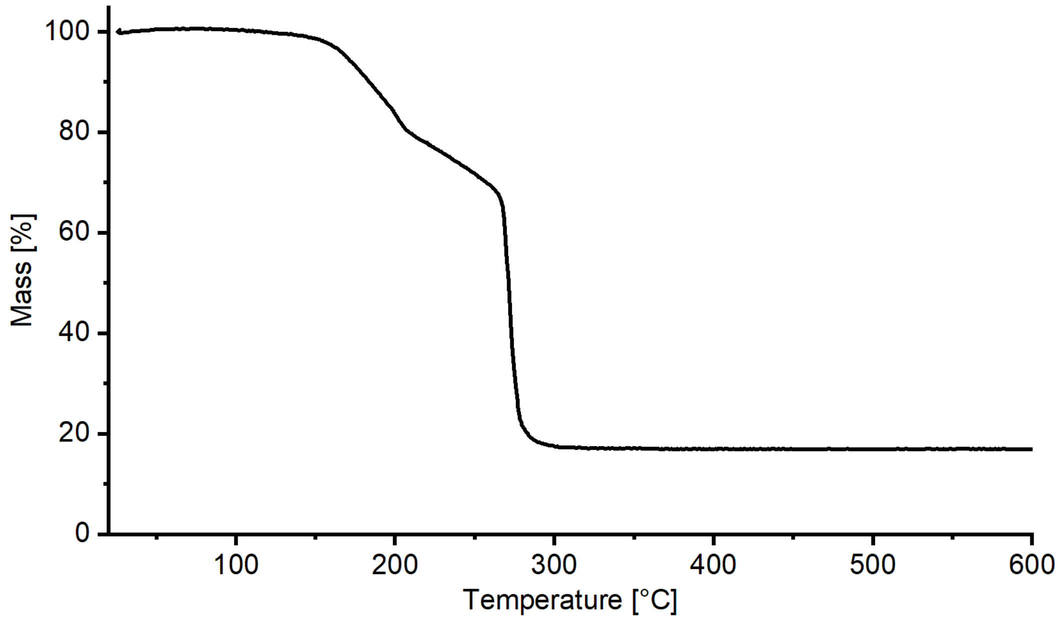


**Figure S6:** Thermogravimetric analysis of the organic-inorganic polymer. The polymeric binder of the organic-inorganic polymer is decomposed between 160 and 330 °C.

## References


[1] R. Paschotta, *Field Guide to Lasers*, SPIE, **2008**.

[2] M. Özkan, S. G. Hashmi, J. Halme, A. Karakoç, T. Sarikka, J. Paltakari, P. D. Lund, *Organic Electronics* **2017**, *44*, 159.

[3] S. Shukla, D. Stanier, M. S. Saha, J. Stumper, M. Secanell, *J. Electrochem. Soc.* **2016**, *163*, F677.

[4] J. Luo, R. Pohl, L. Qi, G.-W. Römer, C. Sun, D. Lohse, C. W. Visser, *Small* **2017**, *13*, 1602553.

[5] J. Hu, M.-F. Yu, *Science* **2010**, *329*, 313.
[6] V. Tasco, M. Esposito, F. Todisco, A. Benedetti, M. Cuscunà, D. Sanvitto, A. Passaseo, *Appl. Phys. A* **2016**, *122*, 280.
[7] J. D. Fowlkes, R. Winkler, B. B. Lewis, M. G. Stanford, H. Plank, P. D. Rack, *ACS Nano* **2016**, *10*, 6163.
[8] A. Botman, J. J. L. Mulders, C. W. Hagen, *Nanotechnology* **2009**, *20*, 372001.
[9] E. U. Donev, J. T. Hastings, *Nano Lett.* **2009**, *9*, 2715.
